# “Even though they insult us, the delivery they give us is the greatest thing”: a qualitative study contextualizing women’s experiences with facility-based maternal health care in Ethiopia

**DOI:** 10.1186/s12884-022-04381-z

**Published:** 2022-01-14

**Authors:** Ashley Hagaman, Humberto Gonzalez Rodriguez, Clare Barrington, Kavita Singh, Abiy Seifu Estifanos, Dorka Woldesenbet Keraga, Abiyou Kiflie Alemayehu, Mehiret Abate, Befikadu Bitewulign, Pierre Barker, Hema Magge

**Affiliations:** 1 Department of Social and Behavioral Sciences, Yale School of Public Health, Yale University, 60 College St, New Haven, CT 06510 USA; 2 Center for Methods in Implementation and Prevention Sciences, Yale University, New Haven, CT USA; 3 Department of Health Behavior, Gillings School of Global Public Health, University of North Carolina at Chapel Hill, 135 Dauer Dr, Chapel Hill, NC 27599 USA; 4 Carolina Population Center, University of North Carolina at Chapel Hill, 123 W. Franklin St, Chapel Hill, NC 27516 USA; 5 Department of Maternal and Child Health, Gillings School of Global Public Health, University of North Carolina at Chapel Hill, 135 Dauer Dr, Chapel Hill, NC 27599 USA; 6 Department of Reproductive, Family and Population Health, School of Public Health, Addis Ababa University, Zambia Street, Tikur Anbessa Hospital Building, Lideta Sub-city, Addis Ababa, Ethiopia; 7 Institute for Healthcare Improvement, Addis Ababa, Ethiopia; 8 Institute for Healthcare Improvement, Boston, MA USA; 9 Bill & Melinda Gates Foundation, Seattle, USA

**Keywords:** Maternal health, Healthcare satisfaction, Childbirth, Healthcare services, Qualitative, Ethiopia

## Abstract

**Background:**

Globally, amidst increased utilization of facility-based maternal care services, there is continued need to better understand women’s experience of care in places of birth. Quantitative surveys may not sufficiently characterize satisfaction with maternal healthcare (MHC) in local context, limiting their interpretation and applicability. The purpose of this study is to untangle how contextual and cultural expectations shape women’s care experience and what women mean by satisfaction in two Ethiopian regions.

**Methods:**

Health center and hospital childbirth care registries were used to identify and interview 41 women who had delivered a live newborn within a six-month period. We used a semi-structured interview guide informed by the Donabedian framework to elicit women’s experiences with MHC and delivery, any prior delivery experiences, and recommendations to improve MHC. We used an inductive analytical approach to compare and contrast MHC processes, experiences, and satisfaction.

**Results:**

Maternal and newborn survival and safety were central to women’s descriptions of their MHC experiences. Women nearly exclusively described healthy and safe deliveries with healthy outcomes as ‘satisfactory’. The texture behind this ‘satisfaction’, however, was shaped by what mothers bring to their delivery experiences, creating expectations from events including past births, experiences with antenatal care, and social and community influences. Secondary to the absence of adverse outcomes, health provider’s interpersonal behaviors (e.g., supportive communication and behavioral demonstrations of commitment to their births) and the facility’s amenities (e.g., bathing, cleaning, water, coffee, etc) enhanced women’s experiences. Finally, at the social and community levels, we found that family support and material resources may significantly buffer against negative experiences and facilitate women’s overall satisfaction, even in the context of poor-quality facilities and limited resources.

**Conclusion:**

Our findings highlight the importance of understanding contextual factors including past experiences, expectations, and social support that influence perceived quality of MHC and the agency a woman has to negotiate her care experience. Our finding that newborn and maternal survival primarily drove women’s satisfaction suggests that quantitative assessments conducted shortly following delivery may be overly influenced by these outcomes and not fully capture the complexity of women’s care experience.

**Supplementary Information:**

The online version contains supplementary material available at 10.1186/s12884-022-04381-z.

## Introduction

The past decade has evidenced effective approaches to reducing maternal and neonatal mortality in settings with resource strained healthcare infrastructure by increasing access to facilities, services, and life-saving interventions [1, 2]. Consequently, global maternal mortality has remarkably declined [1, 3], though inequities persist particularly for disadvantaged populations [4]. As maternal and child healthcare access has improved in low- and middle-income countries (LMIC), attention has turned to improving services with the provision of high quality, respectful care [3, 5–7]. This is particularly important given that increasing access and demand for quality facility-based maternal health services is a core determinant of positive maternal outcomes. Recent multi-level, systems-integrated interventions have demonstrated some success with increasing service coverage, but still have limited impacts on perceived positive experiences with care [8–13]. Specifically, one multi-country investigation found that health service users in 12 LMIC countries had low expectations of care quality, likely resulting in lower demand for higher quality services and inflating satisfaction ratings [14]. More work is needed to further understand how pregnancy and childbirth services are experienced, how satisfaction is articulated and evaluated, and, most importantly, how these findings can improve care provision to optimize quality and subsequent satisfaction and drive future service use for mothers and children [8].

The World Health Organization, building from Donabedian and Hulton’s frameworks [15, 16], proposed a multi-dimensional framework for maternal and newborn quality of care [17]. Recent systematic reviews of qualitative studies of maternal healthcare satisfaction [6, 18] highlight important determinants of satisfaction and demonstrated the utility of qualitative work to uncover the important nuances and descriptions of poor-quality care, illuminating the voices of women to propel change. The authors highlight needs for further research including a need to assess the relative strength of various determinants and further investigations into contextual histories of women’s care navigation to help prioritize recommendations and improvements for maternal health services [7, 19–23]. Importantly, descriptions of experiences of (in) equitable and people-centered care remain thin, despite these domains remaining important drivers of care satisfaction and future healthcare seeking decisions among women. Within these, effective communication, respect and dignity, and emotional support have been less articulated in the literature. Instead, most work has focused on mistreatment and abuses (physical, verbal, and emotional) and health systems limitations in the provision of care (e.g., available resources, staffing, adequate referral, facility quality, etc) [23, 24].

Ethiopia made substantial progress towards several Millennium Development Goals, including reducing under-five mortality and improving maternal health (Goals 4 and 5) [25–29], and recent multi-level healthcare quality improvement initiatives have demonstrated early promise in their efficacy [8, 30, 31]. These initiatives respond to the complex difficulty of improving maternal health service utilization indicators including antenatal care visits, facility-based births, and important follow up postpartum care. In the 2019 Demographic and Health Survey, 52% of women did not deliver at a health facility and less than half (43%) received the recommended number of antenatal care visits [26]. In a qualitative study in four Ethiopian regions (Amhara, Tigray, Oromia and SNNPR), Kaba et al. found that the home environment was prioritized as a safe and comfortable space, affording women important experiences of positive birth-related rituals [32]. This was preferred vis-à-vis health facilities, where births were seen as medicalized, problematized, and access to important home-based rituals were denied. Other qualitative studies demonstrate women’s preferences for home deliveries in the context of structural barriers to accessing facilities and their poor-quality services with limited supplies [33, 34]. Shiferaw et al. found that women preferred the care provided by traditional birth attendants, felt facility-based delivery was unnecessary, and had previous negative experiences in health facilities [35]. While these studies highlight important barriers to care, there is limited research illuminating what satisfactory care means, under what contexts, and how existing expectations and relationships shape maternal perceptions of quality. We seek to fill this gap by presenting birth narratives from women who delivered in government health facilities in Ethiopia. The purpose of this study is to describe women’s experiences, perceived satisfaction, and the contextual events that shape expectations with her maternal health care services.

## Methods

### Study setting

Ethiopia is a geographically and culturally diverse country split into administrative units including Regions (generally with a referral hospital), *Zones* (with at least one general hospital), *Woredas* (with a primary hospital and on average five health centers), and *Kebeles* (with at least one health post). The government health system in Ethiopia is a decentralized model. Health posts (HPs) are the smallest service unit staffed by a government salaried Health Extension Workers (HEWs) and provide basic health services. HEWs are responsible for providing healthcare and education related to family health, vaccination, infectious disease prevention, and select curative services. HEWs can be a pregnant woman’s first and most common interface with the health system. HPs have an affiliated health center (HC) that provide basic emergency obstetric and newborn care [36]. HCs have an affiliated primary hospital that provides comprehensive emergency obstetric and newborn care [37]. Affiliated HCs, HPs and a primary hospital form a Primary Health Care Unit that collaborate to deliver services and, aggregate and share health information. This study was conducted in the regions of Oromia and Southern Nations, Nationalities, and Peoples’ (SNNP). While Amharic is the national language, over 70 languages are spoken throughout Ethiopia. In predominantly rural Oromia and SNNP, estimated literacy rates among women are 48 and 53% [38], respectively. The median age of marriage is 17 and 18 years, and the age of first birth is 19 and 20 years for women in Oromia and SNNP, respectively. Ethnographic work illuminates the complex aspects of the cultural context, agency, aspirations, and expectations of motherhood across Ethiopia. Marriage and motherhood are valued sources of feminine identity and often, a requisite to attain social respect and acceptance. However, vis-à-vis rapid social-economic shifts, a woman’s transition into motherhood is contested with new emerging modern ideals [39].

### Study design and sampling

This study was part of a mixed-methods program evaluation of a district-wide health systems quality improvement intervention implemented by the Institute for Healthcare Improvement (IHI) Ethiopia in partnership with the Ethiopian Ministry of Health. This qualitative study sought to understand how mothers perceived and described their experiences and satisfaction with MCH services received at government facilities. The intervention targeted health staff and used a woreda-wide systems-embedded quality improvement (QI) approach to provide woreda leaders and health facilities additional clinical training in MHC best practices, QI training and coaching of QI collaboratives to identify quality gaps and implement locally derived and cost-effective solutions and provide data quality improvement assistance for the health information systems. Detailed information related to the nationally integrated intervention is published elsewhere [8, 30]. Qualitive data in this analysis was generated from four baseline study Woredas collected between March and April 2018 prior to the invention’s full implementation. In consultation with local health leaders and study staff at each site, we purposively identified and selected one hospital and HC in each woreda that best reflected the catchment area’s patient burden, service quality, staffing, and challenges (e.g., supply chain) to achieve variation in birth experiences.

Childbirth care registries were used to purposively sample five mothers per facility. Our purposive sample frame was theoretically derived to maximize participant variation by parity and age, as we expected that these factors influenced a woman’s care experience. Given the hilly terrain and limited formal roads, facility staff, HEW, and/or community guides assisted the research team to find and approach mothers for consent. Inclusion criteria were being a woman aged 18 to 45 years who lived in the selected study woredas and delivered a live newborn at government health center or hospital participating in the IHI intervention two to six-months before their interview.

Four female research assistants (RAs) with graduate level training in public health, conducted the interviews. The RAs were based in Addis Ababa and unaffiliated with the health facilities in the study sties. RAs received training from Ethiopian (ASE, DWK) and US-based (AH, HGR, CB) investigators with expertise in qualitative research. For example, RAs explored their own positionality and the power-dynamics that may exist with participants. For example, two RAs that were not mothers opened their interviews by explaining that they had never experienced birth or motherhood, positioning the participant with the power and expertise for them to learn from. During piloting, RAs provided critical input on engaged data collection and communication, focusing on specific strategies to explain the study and begin the conversation to encourage participant comfort and positioning them as an expert of whom we are learning from. Other rapport building efforts included the female RAs practicing active listening and asking questions to help engage and facilitate dialogue with participants and be responsive to their local context and styles of communication. Interviews were also conducted in and around their home in privacy, where they felt more comfortable to discuss their healthcare experiences without the near presence of clinicians. Alongside IHI project coordinators and local investigators, RAs (two in each region) engaged with research sites to receive formal health system and community approvals. Thereafter, RAs met with health facility staff and reviewed facility birth registries to create a sampling list of eligible participants. Participants selected the language for the interview. Interviewers in Oromia conducted interviews in Amharic and Afan Oromo, languages that were spoken by the RAs. Interviewers in SNNPR spoke Amharic and used interpreters to interviews participants in Wolaitaa, Dorze or Gamo as they did not speak those languages fluently.

Semi-structured interviews (see Supplement for interview guide) elicited mothers’ experiences of their most recent pregnancy and delivery, focusing on various domains of healthcare satisfaction including staff interactions (communication, skill, trust), services (type, quality, privacy), and facility (cleanliness, equipment, distance) informed by the Donabedian Framework [15]. To contextualize expectations for healthcare services and subsequently, healthcare satisfaction, we explored women’s past delivery experiences, her recommendations for service quality improvement, and influences in her social and community network that shaped her use of government health facilities. We assessed perceived satisfaction by asking participants if they would use the government facility in the future and/or recommend MHC services to women they knew.

Participants provided verbal informed consent to participate in an audio-recorded interview. After each interview, RAs wrote structured field notes on interview characteristics, key themes and reflected on their immediate reactions and recommended foci of subsequent interviews based on information saturation. RAs and the study team regularly debriefed after each interview. Interviewer audios were first transcribed, verbatim, in their original language. Subsequently, transcripts not conducted in Amharic were translated from their original language to Amharic, and then translated to English for analysis. This research was ethically approved by the University of North Carolina at Chapel Hill’s Institutional Review Board and obtained a program evaluation waiver from the Ethics Committee of Ethiopian Public Health Association. All methods were carried out in the protocol outlined in the ethical approval application.

### Data analysis

We used an inductive analytic approach to iteratively analyze field notes and transcripts [40]. Data analysis began during fieldwork, with fieldnotes and team debriefs via conference calls. Informed by field notes, team debriefs facilitated discussions and reflections of immediate observations and impressions as well as domains and emerging themes to assess saturation. Following data collection, we conducted a rapid preliminary analysis of field notes and key observations from team debriefs. In-depth analysis began after all interview transcripts were translated to English. We developed a codebook informed by our preliminary analysis and an initial review of transcripts. Next, we systematically coded transcripts using ATLAS.ti (v8), first double-coding single transcripts and meeting to address coding disagreements and subsequently update code definitions. We did this iteratively until no new disagreements were identified. Half the transcripts were double coded by [BLINDED] and [BLINDED], and the remaining were single coded. Inductive and deductive codes applied and followed by axial coding to relate themes to satisfaction domains. We then wrote delivery experience narrative summaries for each transcript using a matrix. Lastly, we broadly categorized, compared, and contrasted maternal healthcare processes, experiences, and narratives around satisfaction with matrices.

## Results

In total, we interviewed 41 participants: 20 in Oromia and 21 in SNPPR. In Oromia, one interview was conducted in Amharic and 19 in Afan Oromo. In SNNPR, five interviews were conducted in Wolaitaa, 12 in Amharic, three in Dorze, and one in Gamo. Participant details can be found in Table 1. The average age was 28 years and nearly one quarter of women were first time mothers. Among multiparous participants (*n* = 31), the average number of live births was about three and nine had a prior home birth. Nearly 47% of participants could not read and write.

**Table 1:** Participant Demographics Characteristics (*N* = 41)

Characteristic	Total
**Participants**	41
**Age,** mean (range)	27.9 (18–42)
**Household size,** mean (range)	5.4 (3–10)
**Education**	
Can read and write (%)	22 (53.7)
**Religion**	
Muslim	8
Orthodox	16
Protestant	17
**Births**	
Age at first birth, mean (range)	21.3 (17–31)
Live births, mean (range)	2.9 (1–9)
**Parity**	
Primiparous (%)	10 (24.4)
**Prior home birth (%)**	9 (29)
**Health Facility Visits,** mean (range)	3.9 (1–7)

**Fig. 1:**
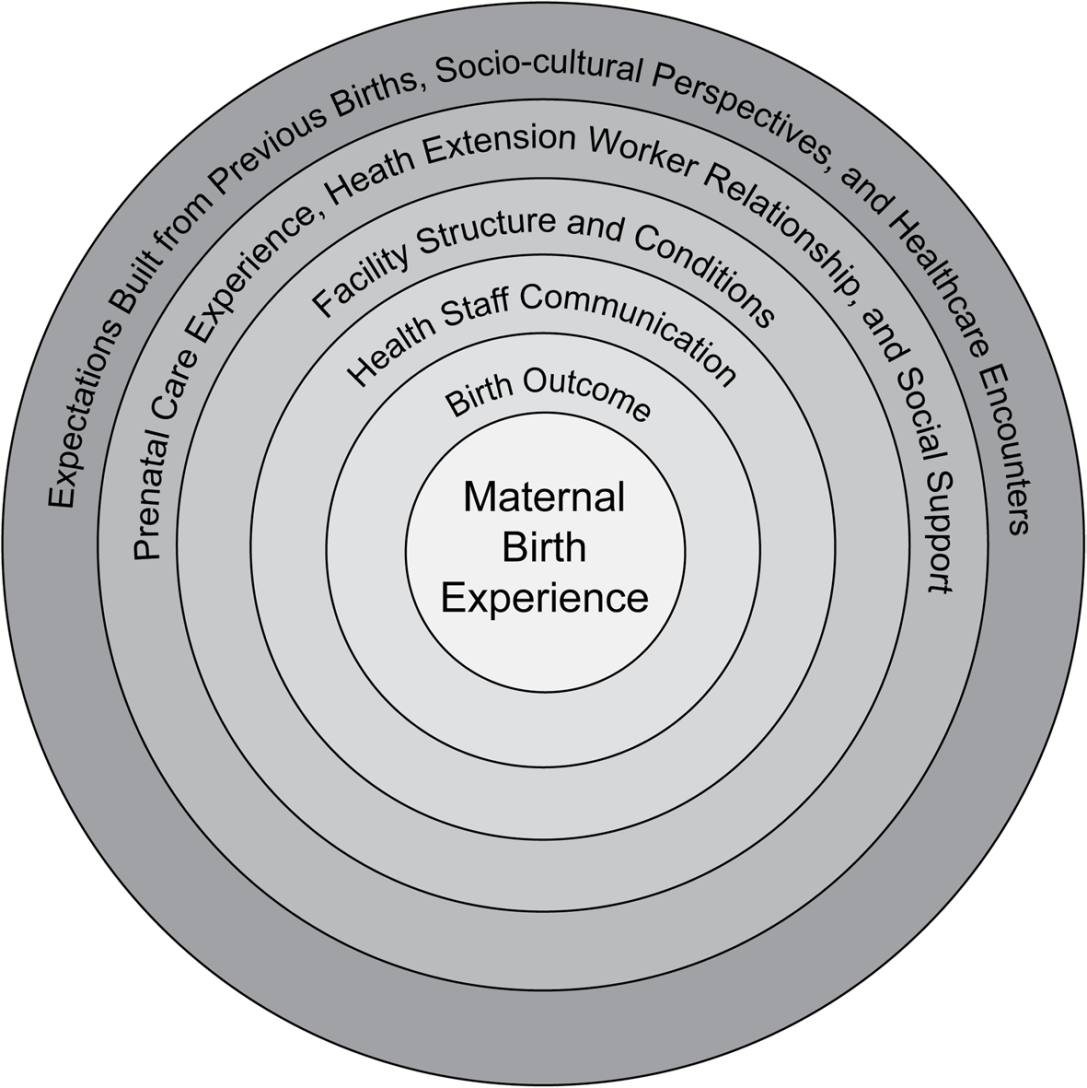
Factors shaping perceptions of maternal satisfaction and quality of childbirth care

We first present women’s descriptions of MHC experiences and then describe the complex array of experiences that built women’s expectations for and reactions to maternal health services. We found that a woman’s appraisal of her birth experience is largely contingent on these expectations and may be perpetuated by tolerance for poor quality care when women are not adequately engaged by their providers. Our results are visualized in Fig. 1, emphasizing the contextual importance of not only direct birth-related determinants of childcare delivery services (e.g., maternal/newborn survival, health staff communication, and facility conditions), but other determinants more indirectly affecting her experience, including her prenatal care and prior births, social learning related to childbirth, and the authoritative knowledge and culture of the healthcare environment. We conceptualize our findings in a circle, rather than a linear diagram, to emphasize the inextricable impacts these experiences and expectations bring to a mother’s appraisal of her care.

### Overall appraisal of MHC experience

Women who described relatively uncomplicated births and delivered healthy newborns generally said their MHC experience met their expectations (e.g., maternal and newborn survival) but expressed ambivalence with the services they received. Care experiences and quality were generally framed as the technical provision of healthcare services. While women reported gratitude and relief following mother-infant survival, secondary aspects that influenced their experience included provider communication and facility amenities. Common themes in descriptions of poor experiences with care included perceived neglect and long delays for care, poor rapport with health providers, and health system constraints. Women’s experiences were enhanced by supportive communication and behavioral demonstrations of commitment from health staff. We first describe the primary drivers of women’s expectations, and then describe various nuanced factors (e.g., individual-level, facility-level and external factors such as social support and economic resources) that shape her experience.

### Survival as a seminal expectation

In their delivery narratives, women expressed survival, their own and of their newborn, during delivery to frame her birth experience. Nearly half of mothers spontaneously said surviving birth delivery influenced their general satisfaction with their delivery care. Mothers expressed gratitude to the facility and its providers following a safe delivery, even when describing negative experiences, such as neglect and verbal abuse, including ridicule and threats. Despite being insulted or threatened, some of these women still felt gracious for services because they prioritized a safe delivery,

“Even though they insult us, the delivery service they gave is the great thing. They treat us [at the hospital]. Below God, they save us from death. But we became angry when they insult us.” (106, 18 years old, first-time mother, Oromia)

The woman quoted above uses a ‘we’ statement, extrapolating her own experience as a common one. Another participant stated: “I say my child [is] saved and [I] keep silent”. Some women may have tolerated poor provider treatment because they depended on staff to deliver safely and may have felt vulnerable, scared and without a choice. Mothers highlighted that providers were tasked with facilitating a safe delivery, framing the care they expected from them as technical and unidimensional (e.g., delivery resulting in healthy baby and mother). Provider encounters were described as ‘services’ and ‘procedures’, suggesting transactions rather than collaboration and partnership. Therefore, perceived provider abuse did not seem to affect overall perceptions of satisfaction with delivery services when there was a positive outcome as many prioritized delivering safely over how they were treated.

Relatedly, a focus on survival created ambivalence around poor facility conditions such as lack of drinking water and food. Few facilities had piped water, and women frequently said drinking water was sparse. One woman did not mind the challenge of basic supplies,

“I didn’t mind because I want to be alive and I was begging them to save me and they told me not worry …then they told me I had a son. I was happy … it was a nice room. But there is water problem.” (404, 21 years old, Mother of 1, SNNP)

Ultimately, surviving delivery and having a healthy baby were the most salient factors that informed women’s reports of their experience.

### Factors facilitating positive experiences of care

#### Provider treatment and communication

Provider care and communication were secondary drivers of women’s satisfaction during delivery. Women wanted to feel cared for and emphasized the importance of human connection within their MHC services,

“…a person has to think, care about another person. If a person does not care about another person, then what makes us human? A person has to think about another person, they must be thinking us, caring for us,” (206, 25 years old, first-time mother, SNPP)

Supportive communication was particularly important to women who felt embarrassed displaying physical vulnerability in front of providers that might otherwise be considered socially improper outside the delivery context, as reflected by one participant,

“The doctors and nurses welcomed me in a good way. Even when I was sick, vomiting, and embarrassed, they told me not to [feel embarrassed] …The nurses help a lot…they encourage me to get it out [screaming] and after I gave birth, I felt relief and happiness,” (410, 28 years old, a mother of three, SNNP).

Some women described their appreciation for providers’ patience and understanding during delivery. One mother recalled shouting at providers when her delivery took longer than she expected and worried a prolonged delivery signaled “problems” for her child,

“I was shouting at them. What they were saying and what I was thinking was not the same…I was hating them, I was asking, “Why you are saying, ‘You have to wait for some time?’ I wanted it [delivery] done immediately, but that’s not how it works.” (206, 25 years old, first-time mother, SNNP)

She appreciated her providers’ calm reaction to her stress and that they explained things to ease her worry.

Mothers appreciated when providers routinely monitored or accompanied them throughout their labor and delivery, as reflected by 104, a 40-year-old, mother of four, Oromia, “She [health staff] never forgot me and observed me now and then”. Women felt safe and reassured when health staff frequently physically checked in on them. In turn, many women disliked feeling “lonely”, or alone, for long periods while laboring. In addition to physical presence, women noted that feeling a connection to the health staff, or having some familiarity with them, made them feel safer, happier, and supported.

“Well, the midwives were girls and they would take care of me, they would come and give me support, they would tell me that it would be okay, when you forget also they would call you using pet names and stuff, it was very good. They would say “It’s ok, you are almost done” and they would really make me feel better… Even when I call them in the middle of the night when I have some pain they would come and treat me. They treated me very nicely.” (202, 30 years old, first-time mother, SNNP)

Such authentic human connections, through supportive emotional care, were valued and created trust. Participants also felt more comfortable using MHC services when providers took measures to safeguard privacy and confidentiality during delivery. For example, while some women may have felt uncomfortable delivering alongside other women in facilities with limited delivery space, they generally expected and trusted providers to maintain details of their delivery private from others not allowed in the delivery room. Women described walls, covered windows, and privacy curtains as maintaining privacy during labor. Trust in the health staff, developed through previous encounters, professional integrity expectations, and practices of ‘taking care’, enabled confidence that the details of their delivery would remain private,

“Well, the nurses can keep a secret; they can keep anything a secret. Other people cannot keep a secret. But they [the nurses] have professional responsibility, and they know everything, they (Other people) will say, ‘This person did this and that’, they might say something because it will be new for them. But for the professionals, it is not new. So they can keep a lot of secrets.” (206, 25 years old, first-time mother, SNNP)

Women felt treated poorly when providers neglected or dismissed their concerns during labor. One mother described initial provider judgement and the need to advocate for admission when she arrived laboring,

“Immediately when I reached [the hospital], they started to check me and the doctor asked me, ‘Why did you come? Your time is not reached. You would be around eight to eight and half months’. I told him, ‘No, I know my months’ and the amniotic fluid had broken. Then they saw me again and it was labor time. I gave birth there.” (110, 21 years old, first-time mother, Oromia)

This mother described feeling “shocked” and “disappointed” after being treated this way by a male staff, where she had to defend her presence and need for care. She credited delivering safely to an attentive female provider who took her concerns seriously. Similarly, another woman described the cold transactional care provided by a midwife,

“She only let me in and checked me, that was the boundary of our relationship…She didn’t greet me…she told us to lay down, and move something on our body, and then they told us to leave the room…I don’t believe in her [knowledge], if she gave me some advice, I would believe in her.” (306, 21 years old, first-time mother, Oromia).

As noted earlier, women often differentiated poor interpersonal care and communication from quality appraisal of their services if they had a healthy delivery, as the woman above had. Mothers valued warm interaction and wanted staff to demonstrate their expertise by providing information and advisement. Women recommended improvements in staff interpersonal interactions and privacy (particularly in the hospitals). When asked if women would recommend to friends or other women to use the health facility, they almost exclusively said yes, even if they had a negative experience. This first-time mother reflects this stating,

“If they [other women] ask me, I will advise them to go to health center…Despite their behavior, the doctors have knowledge, I don’t have any doubt in their knowledge.” (306, 21 years old, first-time mother, Oromia).

Another mother (408, age unknown, mother of eight, SNNP) said she didn’t tell other mothers about the negative aspects of her care because, “if I told them, they may not go [to the health facility]”. These women supported their recommendation by elaborating that the safety and emergency services provided in the hospital were worth enduring disrespect.

#### Facility structure and conditions

Physically clean facility conditions supported positive birth experiences. Some women highlighted the access to janitorial services as well as help cleaning themselves after delivery as a reason to deliver at a facility rather than at home. The attention to cleanliness gave mothers comfort, as one described,

“I think what they are doing is an excellent job, especially during labor the cleaning lady cleans everything right away…it makes us so comfortable.” (410, 28 years old, mother of three, SNNP).

Alternatively, women cited poor sanitation, such as soiled beds, floors, and walls, and structures providing inadequate privacy and confidentiality structures as areas that they felt could be improved (thought notably, did not drive overall dissatisfaction strongly). Pungent smells made some women feel sick, made it difficult to eat, and propelled women to quickly leave the facility following childbirth.

“There was bad smell in the room; you couldn’t stay a minute there. Doctors even talked to the janitors about cleaning. They told us it is not good for you and your baby, better to go to your home… I was happy for my safe delivery but I was disappointed by the room quality.” (108, 25 years old, mother of two, Oromia)

Some women felt that poor facility sanitation reduced overall service quality during delivery, “The quality of the hospital was not good because they were not cleaning the bed on which women stayed during delivery” (306, 21 years old, first-time mother, Oromia). Poor sanitation and lack of cleaning staff also extended to the post-natal waiting rooms, where women described blood on the beds and floors. Mothers suggested more cleaning practices, testing, and supplies, such as more mattresses for the birthing beds and ultrasound machines. This too, directly related to transactional care.

The tangible and often mechanical receipt of services played an integral role in the assessment of maternal and neonatal health services. Services tied to comfort and ‘care’ were deeply appreciated, though not expected. For example, when available, women appreciated receiving bedpans, blankets, food, drinks and functional assistance (bathing, assistance using the restroom) throughout delivery. For example, 104, appreciated the privacy and assistance with waste removal, an unexpected service,

“To receive urine, they put something beside you. They leave you alone assuring you to feel free. What could be more comfortable than this! During delivery I was on a high bed and there is a receiver for waste under the bed where it drains. What more than this can she do?” (104, 40 years old, mother of four, Oromia)

Women with higher risk pregnancies also appreciated having access to a maternal waiting home (structures near the health facility typically built in a traditional way, emulating an Ethiopian home to increase comfort for longer stays for high-risk women) as they waited to deliver. Other women appreciated receiving food, drinks, and having their clothes washed. These additional unexpected services, and often the staff that provided them, benchmarked moments of comfort and feeling cared for.

Some women did not seem to expect services to help them throughout the delivery process. Women appreciated this help, which consequently increased comfort and satisfaction with their delivery experience. Interestingly, the lack of these services, however, did not typically diminish a participant’s positive experience with delivery services as many did not expect to receive some of these services to begin with.

#### Social support and satisfaction

Material and social support supplemented women’s experience and subsequent ‘satisfaction’ with MHC, suggesting satisfaction with delivery experience is extended beyond facility factors and providers. For example, friends, families, and neighbors provided mothers with food, clothing, and blankets that were in short supply at facilities during delivery, as described below,

“There was leather on the bed which attracts cold. You feel cold when you sleep on it. There was nothing except that leather. [There was no blanket], I took it [blanket] from my home. Additionally, my husband sent me [another] blanket from home.” (– 307, 31 years old, mother of two, Oromia).

Relatives also provided women with food, medicine and clean clothes, supplies they did not anticipate receiving at a health facility, reflecting the central role of external support during the delivery experience. Women expected staff to provide medicine and medical services but not necessarily any additional provision of comfort. Women expected support such as encouragement, accompaniment, or assistance bathing from family and friends throughout delivery, though not necessarily from staff. When birth companions and family engagement was experienced during labor and delivery, women noted their gratitude, suggesting that it was unexpected that it would be allowed. For example, 104, appreciated having her *agabaatte* (husband’s sister-in-law) support her in the delivery room,

“I felt great pleasure for her [agabaate] coming in. The midwife allowed my “agabaatte” to come in after seeing that I was becoming tired. While I was in misery, I grasped her and pushed the baby out with effort. I felt as if she inserted her hands into my body and took out the baby” (104, 40 years old, mother of four, Oromia)

However, women varied on their ability or desire to have family, friends, or neighbors present during delivery. For example, many women believed staff did not permit others in the delivery room, “Nobody was allowed to enter there,” said, 106, 18 years old, a first-time mother, Oromia, who did not bother asking, believing it was against the rules. 106 is from a distant community and had no family support throughout pregnancy and delivery. She relied on a neighbor to support her throughout labor,

“She was staying with me when there were no doctors with me but when doctors came, she stayed outside. She helped me greatly after I gave birth too, preparing coffee, hot drink like ‘*atmit’*, preparing food for me and things like that.”

Other women did not want relatives or neighbors present, even if permitted, viewing it as impractical or unnecessary. “Why would they go in when there are nurses? There are three or four nurses, so they will not allow them, and I don’t want them there either”, said 201, 29 years old, mother of three, SNNP, again echoing the perception that staff did not permit others in the delivery room. Some women did not want others in the delivery room because they felt embarrassed, ashamed or uncomfortable delivering in front of others. Other women distrusted relatives and neighbors with their privacy. Therefore, variations in preferences for social support alongside different perceptions on what staff would allow created differences in women’s perceptions of the quality of care they received. Some wanted more privacy and the belief that others were not allowed in the delivery room helped maintain their privacy, while others assumed their companions would have remain outside, and their preference was not considered when assessing their perceptions of the quality of care. Material and social buffers then, play important and complex roles in perceived quality of care, contingent on what women expected and understood a priori.

#### Health extension workers created opportunity for increased quality care and satisfaction

HEWs were often a mother’s s first facilitator for using government health services for MHC, particularly in rural areas, providing initial education about pregnancy and childbirth and advising on what services she can access. Thus, HEWs set mothers’ initial expectations for anticipated services, particularly that a facility delivery would afford them a safer and more hygienic childbirth. One mother explained,

“She [HEW] teaches us how our health will get improved if we visit the health center. That my baby will be healthier and I will be more relaxed with my body…I will know how my baby is doing inside.” (406, 25, mother of five, SNNP)

HEWs also provided education around warning signs and complications to propel women with more autonomy in their care. The relationship HEWs developed with mothers contributed to women’s satisfaction with delivery by providing multiple forms of support and advocacy that complemented the services provided at the facility. For example, 402, 30 years old, mother of three, SNNP, described the HEW as someone who, “could find a solution for any problem that existed.” HEW support in labor and delivery was described as both instrumental and emotional. HEWs were frequently the first point of contact and played a key role in facilitating access to and from HCs and hospitals and helping women quickly obtain or navigate care upon arriving at facilities, as again illustrated by 406,

“She came to the health center and asked me how I was doing. Then she asked why I didn’t call her and, when I left the health center, she took me to my home paying for the motorcycle. She told me to take the things given by the government, like oil and bread flour.”

This narrative highlights a disconnect between what HEWs expect to do for mothers (and the resources they know are available to them) and what resources and support mothers understand is available to them, suggesting important equity gaps.

HEWs also emotionally supported women throughout delivery. 210, 18, a first-time mother, SNNP, liked that HEWs accompanied women to the clinic so they did not have to go alone, “She is a good person. She goes with us. We follow her teaching.” Other women appreciated when HEWs accompanied women throughout delivery, fulfilling a support role not often given by the health staff. HEW’s encouraged women to relax and aided with pushing in the delivery room. One mother felt good knowing that the HEW did not leave her site during delivery. Others described how the HEWs brought them blankets during delivery. Disparities in HEW relationships and support existed, as some women did not engage with HEWs beyond a couple limited interactions, thus not affording them a buffer if there was insufficient emotional support from health staff.

### Past experiences shaped delivery care expectations

In addition to the individual, facility and social support factors that influenced satisfaction, women’s narratives of satisfaction with their latest birth were inextricably linked to their past births and antenatal care. Five women that had delivered previously in a government health facility reported positive past experiences using healthcare services. 204, 26 years old, a mother of two, delivered her second child at the same health center as her first because she felt staff went beyond their scope of work to care for her,

“[This health center] is really good. When I was giving birth to my first baby, I went in the morning and when it got to lunchtime, I asked him [the staff] not to end his shift. He was a night duty so he should leave at 9:00 in the morning, but he stayed for me without even eating his breakfast, when he said there is another person on duty and he had to leave, I asked him to stay. So, he stayed [with me] without eating lunch. He stayed with me until I gave birth.” (206, 26 years old, mother of two, SNNPR)

Feeling ‘cared for’ and experiencing health staff committing themselves to their care encouraged women to return for future MHC services. The relationships these mothers established with the healthcare providers (e.g., midwife, HEWs, etc.) helped build trust and familiarity with the care processes and procedures of childbirth. Their previous safe delivery made them more confident of a subsequent safe childbirth, reinforcing expectations that delivery at the facility would result in a healthy infant.

Multiparous mothers with a prior homebirth and a facility delivery naturally contrasted their experiences, underlining what mattered most: maternal and infant survival. A mother of five described,

“I had near death experience because the umbilical cord was not cut. The baby was crying laying on the floor and the person who was supposed to cut the umbilical cord lost the blade and I was scared. I didn’t want that experience again, so I decided to go [to the health center].” (406, 25 years old, mother of five, SNNP)

She then recounted after her delivery at the facility,

“They [health staff] took good care of him [her son]. They cleaned me and transferred me to another bed, and I was happy. If God gave me another child, the health center is my choice…they [the health staff] are doing a great job.”

Facilities brought feelings of safety and security for this mother, and she expected that her baby’s health would be ensured. These women said delivering at a health facility afforded access to trained medical staff that could provide life-saving procedures if they experienced a medical emergency and provide medications to alleviate pain, as described by this mother:

“They gave me an injection [medication to alleviate pain]. When I gave birth at my home, I was feeling sick. I was feeling stomach sickness for one week at that time. But when I gave birth at hospital, they gave me syringe, medicine, and glucose. So, it was [a] better [birth experience] here in hospital” (303, 39 years old, mother of three, Oromia)

While most women highlighted the safety and reduced fear of facility deliveries, they also described comfort and reduced stress with home delivery, which included receiving family support, trusting those present, and the allowance for preferred practices (e.g., standing during labor). Women with prior homebirths liked being surrounded by family, receiving food and material comforts (e.g., clean blankets), and, importantly, privacy. Facility deliveries carried the expectation that privacy, traditional preferences (e.g., standing), and warm home comforts would not be afforded to them. This, however, did not appear to reduce their satisfaction with facility deliver because the expectations were adjusted.

Antenatal care (ANC) visits at health facilities and community-based support received from the government health system through HEWs, also generated expectations for the current delivery. The interpersonal relationships defined and developed throughout prenatal care played a complex role, both creating trust and frustration. ANC visits allowed a woman to learn how to efficiently engage with the health system to optimize their care. For example, a mother of one initially received ANC care at the closest health center, but switched to the hospital,

“Because doctors helped you in good manner. The doctors in the hospital treated you in good manner. But, in the health center, health workers even insult you.” (302, 25 years old, mother of one, Oromia)

Experiencing disrespectful care propelled this mother to find providers that treated her better and, after feeling treated well in her ANC care, she expected kind treatment during delivery. During ANC, women also learned to bring appropriate supplies and documents (blankets, food, and health card) to improve their experience. Several women said physically carrying their health card was a necessity for both efficient care and proper treatment. Failure to have a health card may mean not receiving care at all, or, certainly, receiving exasperation, rudeness, and a longer wait. ANC, thus, allowed women to accumulate patient capital and agency – women with few ANC visits on her health card wielded less power in the delivery room, stating that they may be blamed or mistreated because they did not come for care.

Women expected health staff to “do something” during ANC to feel reassured about their baby’s health, referring to tangible testing procedures including ultrasounds, blood, urine, and blood pressure tests, and checking the position of the baby. A first-time mother from SNNP explained,

“knowing that my baby is in good health, also that my health [is good made me happy]. And I believe that nothing is more important [to know] than this (404)”.

Women not provided testing expressed frustration and providers, “did nothing” for them. The phrase “did nothing” was common across many interviews. Women seemed to prefer and expect a tangible test above and beyond receiving education and information. Many mothers indicated that they never wanted or needed to ask the staff any questions, perhaps indicating their trust in the “tests” and staff’s to detect any issues. When women *were* treated with kindness and respect, they were overwhelmingly appreciative, suggesting that kindness and respect were not necessarily expected, as illustrated by one woman 410, who described and compared her experiences with a male doctor and a female clinician (specific clinical title unknown),

“At first it was a woman who checked me and second round it was a man, all of them knew their job very well but the man was more knowledgeable. …the female didn’t ask a lot of questions but the man was a doctor, and asked me questions a lot…. he asked if things are favorable at home, like the food and if I drink a lot of water, which will be very good for myself and the baby…He also gave advice to keep stress away because the stress will hurt the baby so much…. I was comfortable even though he didn’t speak my language (woletiega) and I was worried I might not understand what he is saying, but I speak and listen to Amharic, so it was nice of him to think of his patients because he wants us to understand.” (410, 28 years old, mother of three, SNNP)

As with delivery, staff kindness, patience, and dignified communication could forgive infrastructural barriers and poor-quality care, like longer wait times. For example, 403, 30 years old, mother of two, SNNP, said, “there is a long line, but they examine us properly”. Other women stressed that when staff were able to give them ‘human dignity’ (*kibir*) throughout ANC, they were quite happy. One first time mother, after stating her initial fear from rumors that the staff were rude, relayed,

“she was treating me very nicely, she was very playful and making me laugh. She was comforting me saying there is no problem, it was very nice. (202, 30 years old, mother of 1, SNNP)”

Another primiparous mother said that staff explaining treatment options for possible concerns was in itself empowering,

“If for instance they say your blood is low, and I need to eat and drink to increase it. It’s nice when they tell me before it can cause a problem, if your weight is low then you are told it has to be like this then you eat to increase it. When they say, this can lead to be a problem to the baby, its blood pressure might go high or low and that dangerous for the baby so you to check your self and take whatever is needed, it’s really nice. It’s really nice when you are told you need to do this because you need it. (110, 21 years old, mother of 1, Oromia)”

ANC therefore set expectations for what is ‘normal’ or routine MHC care, such as infrastructural expectations (e.g., wait times), service expectations (e.g., ultrasounds and testing), and type of staff treatment (in some cases straightforward and impersonal, while in others, empowering, respectful, and educational). ANC also served as a critical time when mothers developed relationships with the health system itself prior to delivery. These ‘benchmarks’ of expectation and developed relationships follow mothers into the delivery, shaping their expectations for birth and, ultimately, their subsequent satisfaction.

## Discussion

This study elicited birth narratives and perceptions of care from 41 women that recently delivered in government health centers or hospitals in two Ethiopian districts. For all women in the study, the seminal driver of a positive birth experience was maternal and infant survival and safety. Deliveries with healthy outcomes, especially their infants, were nearly exclusively described as ‘satisfactory,’ when asked directly, but these women went on to describe instances of provider disrespect, neglect, and poor facility quality (e.g., no running water, overwhelming odor, etc). Although women express ‘satisfaction’, they highlight simultaneous negative experiences (e.g., anger, mistrust, neglect) as well as empowering ones (patient communication, behavioral expressions of caring, and social support). Women described maternal and infant survival as a priority over poor experiential quality care and abuse and mistreatment. Material and social support from friends, family, neighbors, and HEWs supplemented women’s experience and subsequent appraisal of their MHC, particularly when they did not receive, or expected to receive, this type of support from health facilities and its staff. We highlight that women described their experiences of their latest birth contextually with other prior events, such as ANC healthcare encounters and past home or facility-based births. This expectation benchmarking shapes their expectations for birth and, ultimately, their subsequent appraisal of their experience.

Previous studies have highlighted the importance of maternal and infant survival in healthcare satisfaction, particularly in low- and middle-income settings where under-five mortality remains relatively higher [41]. Cham et al. (2009) found that maternal survival following an obstetric emergency was associated with satisfactory care, even, in some cases, when the newborn did not survive [42]. In line with this, Roder-DeWan et al. highlight that low expectations drive high care quality assessments [14]. Alternatively, a 2015 systematic review focused on LMIC found that infant survival and birth outcomes were a ‘minor determinant’ (e.g., reported in fewer than five studies) of maternal satisfaction with healthcare. In our study, women highlighted this as a deeply important condition for satisfaction, dismissing, excusing, or ignoring disrespectful or abusive care if healthy birth outcomes were met. This underlines that when expectations of care are low, the cultural capital given to health providers and birthing facilities silences women from communicating and engaging in their own care beyond expecting the bare minimum [43]. O’Donnell et al. highlighted that women may not expect or advocate for respectful care, fearing that confronting or disagreeing with staff so may incur poor outcomes [44]. Similar findings were highlighted in Rwanda where despite improvements in quality of care and neonatal mortality, patient satisfaction was not significantly improved [11]. Beyond the explanation that O’Donnell proposed, the authors also highlight that the health systems intervention may have not had adequate focus on improving dignity and respect during patient contact. These explanations may be the case in these study sites in Ethiopia, though components of the larger intervention (fully implemented after this data collection) did focus on some specific strategies to increase provider delivery of dignified and respectful maternity care [45]. In concert with this extant literature, our results also highlight that more opportunities may be indicated for more patient education and time for information sharing during healthcare encounters to increase the relative expectations and subsequent power of maternal patients. While women in our study did not describe fear, they did describe ‘remaining silent’ in instances of receiving poor care. Particularly in rural areas (where our data was collected), women may not be afforded opportunities to exhibit agency, and the existing culture of the health system may reinforce disempowerment and complacency with care.

Beyond maternal and newborn survival, mothers noted that interpersonal communication and demonstrations of ‘caring’ by health and facility staff impacted their experience, though not necessarily overall satisfaction. Patience, emotional support, and communication certainly increase positive birth experiences [46], but midwives and other health staff encounter various barriers, both structural and cultural, to implement these elements into their care [47]. The positive aspects of care that mothers highlight are important contributions to the maternal care literature given the focus to date on disrespect and abuse. Other interpersonal aspects of care, such as decision making and active participation in childbirth, were not explicitly highlighted by the mothers in our study. Shakibazadeh conducted a review and found that women in LMIC were less likely to demand and prioritize these aspects of care compared to women in high-income countries [48]. Women may not be empowered enough to be actively involved in their facility-birth. One explanation may be that many women are unable to accumulate the cultural health capital valued by health staff so that they are left with sub-optimal health care relationships [43]. For example, women in our study may not have deployed the verbal competencies, interactional styles, or future-oriented decision-making approaches that providers are trained to respond to. Thus, they experienced less provider communication and fewer opportunities to attain agency in their own care. There is, however, healthcare provider consensus that provider communication and perceived experience of care are important dimensions of high-quality care delivery, and more work is needed by care workers themselves to better implement holistically respectful care [49]. Indeed, Ethiopia has targeted compassionate and respectful care as a core pillar of their Health Sector Transformation Plan, and respectful maternal care trainings are being tested and implemented nationwide with some success [45], as have other interventions in Tanzania [50] and Kenya [51], thought important challenges remain in practice [52, 53]. Mothers in our study noted special quality of care when birth attendants provided them continuous accompaniment, attention, privacy, and support. Supporting the introduction of a birth companion into the labor process was a focus of the larger quality improvement intervention and national maternal quality care initiative [30, 45]. These factors and improvement approaches have been highlighted in other contexts as well [54–56] and while respectful maternity care trainings address the patient communication domains, provider absenteeism and overwhelmed and overburdened systems may continue to make it difficult to pragmatically provide such care [57].

To date, conceptual frameworks of maternal satisfaction focus mostly on point-of-care aspects. While this is undoubtably important, our findings highlight that what women bring to their birth experience, including their previous experience with their antenatal care, previous births, and social exposure to birthing norms and experiences of their social networks all shape expectations. In line with theories related to cultural health capital, the expectations and responsibilities women place on health providers, combined with the expectations placed on them as ‘compliant patients’ by the medical system, privilege the actions and treatment deployed by the more powerful providers [43]. Bradley points to health providers need to maintain power and control, particularly in resource strained contexts with immense pressure to perform well on paper, further contributing to existing inequities in quality care [47]. Considerations of these domains must be incorporated in MCH quality of care frameworks in order to create environments where healthcare systems attend to histories of inequity, discrimination, and other deeply important experiences that women bring with them into the delivery room. This also extends to growing efforts to create patient centered care and derive quality metrics [18, 58–60]. Our results support the need to further develop these valid metrics for more routine use to continuously improve person-centered care.

Our study has limitations. Our findings may not be representative given our purposive sampling of women in two regions of Ethiopia with distinct cultural and geographical contexts. We excluded women that had stillbirths or early neonatal mortality from our study and this limits our ability to reflect on satisfaction among this population. However, our findings may provide transferable insights and ideas that could inform research in other settings. Interview audio, depending on the interview language, was double and sometimes triple transcribed as we conducted data analysis in English. As such, it is possible that cultural and linguistical nuances in communication, such as idioms, tone, etc. were omitted or changed during the translation and interpretation process. Next, the personal characteristics such as class, education, maternity status, age, and cultural background between the participants and interview team may have influenced power dynamics and therefore, how some participants responded to questions. For example, it is possible some participants felt less or more comfortable expressing and sharing their experiences with the research team, particularly if health staff, HEWs, interpreters or others aided the team to find or interview women. Additionally, communities and a mother’s positionality deeply shape what they may feel comfortable sharing in a formal research context. Although our team took community-informed steps to build rapport, create comfort, and ensure confidentiality, some mothers may feel uncomfortable sharing negative experiences or expressing dissatisfaction with MHC services or specific individuals that live and/or work in their community. Future research could elicit maternal experiences with antenatal and postpartum care are warranted, as these perinatal health encounters also contribute to a woman’s perception of her maternal healthcare, as well as her future decision making and influences on her broader social networks.

## Conclusion

Our findings extend the literature on maternal satisfaction in healthcare by highlighting the importance of understanding past experiences and expectations, as well as the social and instrumental resources available to her during the delivery process. Our finding that newborn and maternal survival drove positive reflections on birth experiences, suggests that assessments conducted shortly following delivery may be overly influenced by these outcomes and not fully assess the overall experience. Novel findings, such as the power and agency some women accumulated through antenatal care, examples of empowering patient-provider communication, and the expectations women brought into the delivery room highlight the importance of engaging and assessing quality in maternal health care in multi-dimensional ways in order to understand the complexities of patient experience. Improved assessment of experiential quality as a key healthcare quality outcome may be more relevant that measuring overall satisfaction, particularly in contexts with limited health resources.

## Supplementary Information

**Additional file 1.** Interview guide

## Data Availability

Data is available upon reasonable request directed to the author Professor Clare Barrington (cbarring@email.unc.edu).

## Abbreviations

LMIC: Low and middle-income countries
SNNPR: Southern Nations, Nationalities, and Peoples’ Region
HEW: Health Extension Worker
HP: Health Post
HC: Health Center
QI: Quality improvement
RA: Research Assistant
IHI: Institute for Healthcare Improvement
ANC: Antenatal care visits
MHC: Maternal Healthcare
