# Supplementary material for: “Even though they insult us, the delivery they give us is the greatest thing”: a qualitative study contextualizing women’s experiences with facility-based maternal health care in Ethiopia
Source: BMC Pregnancy Childbirth. 2022 Jan 14;22:31. doi: 10.1186/s12884-022-04381-z (PMC8759250; doi:10.1186/s12884-022-04381-z)
Supplement: Supplementary file 1 — Additional file 1. Interview guide [file 12884_2022_4381_MOESM1_ESM.docx]

**Participant ID**: ______________

**Date:** _____________________

**Interviewer name:** _________________________

**Language of Interview:** _____________________

**Translator (if present):** ___________________

**Woreda**: _____________________

**Health facility: _______________**

Age ______

How many live births have you had?: ________

How many children do you have?: ___________

How old were you when you gave birth to your first child?: _______

Marital Status: _______________ Ethnicity: ____________________

Occupation: ______________________ Religion: _______________

Number of people in household: ___________

Education (highest grade completed): ____________

How many times did you visit this clinic during your pregnancy, including delivery? : ______

Did you receive care at other health centers during your pregnancy? ____Yes ____No

If yes, which health center? ________________________________

PURPOSE: ESTABLISH RAPPORT, ALLOW MOM TO BECOME COMFORTABLE TALKING ABOUT HERSELF

1. **To begin, I would like to know more about you as a person. Tell me about a typical day in your life. From the time you wake up to the time you sleep, tell me about what you do.**

PURPOSE: UNDERSTANDING MOM’S EXPERIENCE WITH PREGNANCY

1. **I would now like to learn more about you and your experiences with your most recent pregnancy. Can you Pick one day in your pregnancy and tell me about it?**

- How did that change later in your pregnancy?
- What, if any, worries did you have?
- What made you feel excited?
- When appropriate, probe if she wanted the pregnancy at the time she got pregnant and how she found out

1. **Can you tell me about any traditions/practices that you follow during pregnancy?**
   - Are there any restrictions on what women can do when they are pregnant? (probing for how this might affect accessing care)?
   - How did these restrictions affect your ability to get care?
2. **What was it like when you returned to your home following birth?**
   - What were some of the things your family/community did? Said? How did it make you feel? .

PURPOSE: UNDERSTANDING WHAT LED MOM TO UTILIZE MATERNAL HEALTH CARE AT HER FACILITY (WHY, WHERE, WHO)

1. **I would now like to learn about your experiences getting healthcare during your last pregnancy. Can you describe how you ended up getting healthcare for your pregnancy at this clinic/facility?**

- Tell me about why you accessed health services for delivery/during your pregnancy?
- How did you select this clinic/facility?
- Who, if anyone, influenced you to get care at the facility (HEW, mother, husband, women’s group, etc)
- What, if any, concerns did you have coming to this clinic/facility?

PURPOSE: LEARN HOW MOM EXPEREINCED MATERNAL HEALTH SERVICES (ACCESS, SPACE, STAFF, BELIEFS ABOUT CARE)

1. **Think about your delivery experience (the day you delivered). Can you walk me through your appointment from the moment you arrived at the clinic until you left.**
   - How long did you wait to get care from the moment you arrived?

- What or who were you waiting for?
- How typical are wait times for you?
- How did that ____ make you feel? (waiting a long time, etc).

1. **ACCOMPANIMENT**: Who if anyone accompanied you to appointments?

- Probe first for the time of delivery/birth and ANC/PNC
- *If accompanied*: what was it like having that person there?
- *If accompanied*: How did this person help or support you? Do you wish they could have helped in another way?
- *If accompanied*: Who decided who would go with you?
- Would you have liked to be accompanied by someone? Tell me more…
- Who would you want to be there with you?

1. **SPACE: I do not know much about the clinic you attended. Can you describe the space where you received services to me?**

- What does it look like? Feel like?
- How do you feel at this clinic/facility?
  - (Probe: (dis)comfort, privacy, curtains, screens, others watching, overcrowding, etc.)
- Tell me about other people that were in the room while you were delivering? Who were these people?
- *If others were present:* Were you asked permission for others to be there?
- How did your experience align with local practices or expectations?

1. **STAFF: Please tell me about all of the different people from the clinic/facility that you interacted with. Who were all the people who provided care to you at the clinic?**

- What did each person do?
- What kinds of things did you like about the staff at the clinic?
  - How were you treated by people at the clinic? (Probe: shame, respect, neglect, humiliation, physical abuse, etc.)
- How often were you asked for your opinion or things you want at the clinic?
- What did you not like about the staff at the clinic?
  - Probe: What happened? How did you feel?
- How confident did you feel in the knowledge and skills of clinic staff? Tell me more…
- Probe for non-clinicians (guards, secretaries, etc)

1. **COMMUNICATION WITH PROVIDERS: Now I would like to talk to you about the way the doctors and nurses explained things to you.**

- In general, how well did you understand what the doctor/nurse was doing during your appointments? (probe if services were explained, if she was confused)
- Were you able to ask the doctor or nurse questions? Tell me more…
- How did the doctor or nurse get permission from you to perform services? How did you feel about this?
- Tell me about how the nurses and doctors spoke to you (supportive, encouraging, harsh/strict)? How did that make you feel?

1. **How do you believe the services you received affected you?**

- Did anything significant change?
- How do you believe the services you received affected your child?

PURPOSE: TO UNDERSTAND HOW MOM’S SATISFACTION WITH HER MATERNAL HEALTH CARE AND FUTURE USE

1. **Tell me about your overall impressions/thoughts with the care you received at clinic/hospital.**

- Tell me about your overall impressions with staff at the clinic/hospital.
- Based on your experiences, would you use these services again? Tell me more about why or why not.
- Would you recommend these services to other pregnant women? Tell me more about why or why not…

1. **How can the staff at the health facility make the experiences of childbirth better?**

- What can the clinic do to improve care for moms?
- What would make you feel more comfortable and want to come back to the clinic?
- What can staff do to improve care for moms?

1. **Thank you for taking the time to share your experiences with me, I really appreciate your time and participation.**
   - Is there anything else you would like talk about more about before we end this interview?

- Do you have any questions or comments for me?
